# Determination of the relationship between parents' health literacy and fever management of their children: A cross‐sectional study

**DOI:** 10.1111/jan.16275

**Published:** 2024-06-17

**Authors:** Dilek Menekşe, Öznur Tiryaki, Nursan Çınar

**Affiliations:** ^1^ Department of Peadiatric Nursing, Faculty of Health Sciences Sakarya University Sakarya Türkiye; ^2^ Department of Midwifery, Faculty of Health Sciences Sakarya University Sakarya Türkiye

**Keywords:** fever, fever management, health literacy, parent, PFMS‐TR, THLS‐32

## Abstract

**Aims:**

The research was conducted to determine the practices of parents regarding the fever management of their children and reveal the relationship between their health literacy (HL) and fever management of their children.

**Design:**

Cross‐sectional study was used.

**Methods:**

This study was carried out with 242 parents. The data were collected using the Parent Descriptive Information Form, Turkish HL Scale‐32 and Parents' Fever Management Scale between September 2021 and September 2022. The data were evaluated with the SPSS program, using percentages, averages, Pearson's correlation and regression analysis.

**Results:**

The mean age of the parents was 31.87 ± 6.59. The parents' mean Parents' Fever Management Scale score was 36.22. It means that parents had high fever management practice. Their mean Turkish HL Scale‐32 total score was 34.43. 51.6% of the parents had a problematic or insufficient HL level. In the cases of fever, 61.2% of the parents stated that they took off the child's clothes, 69.0% measured temperature from the armpit, and 55.4% gave antipyretics according to the doctor's prescription. There is a statistically significant positive correlation between the Parents' Fever Management Scale and Turkish HL Scale‐32. It is observed that 8.2% of the change in parents' fever management is explained by HL.

**Conclusion:**

The study found that with an increase in parents' HL, fever management of their children also increased.

**Implications for the Profession and/or Patient Care:**

Emerging evidence showed that developing parents' HL knowledge and skills could be an option/approach in fever management. It should be a basic nursing skill that to provide HL support to parents.

**Reporting Method:**

This study adhered to the relevant cross‐sectional STROBE (the Strengthening the Reporting of Observational Studies in Epidemiology) guidelines.

**Patient or Public Contribution:**

No patient or public contribution.

## 1 INTRODUCTION

Fever, one of the most common symptoms of illness experienced by children, is a situation that causes extreme anxiety in parents (Wilson et al., 2019). Fever anxiety or phobia continues to play a significant role in the fever management decision‐making process of caregivers of febrile children irrespective of country of birth, living in either metropolitan or regional areas or age and number of children (Arias et al., 2024). Insufficiently informed parents need unnecessary laboratory tests and hospital visits to calm their concerns. However, they may use unnecessary antibiotics and make incorrect and unsafe practices that may pose a danger to the child (Çaka et al., 2015; Gulcan & Sahiner, 2022; Thompson et al., 2020). At this stage, it is important to know the fever knowledge, attitudes and approaches of parents, which can be different in every culture. Parents need to be informed and supported by different methods about fever management. Health literacy (HL), which has been one of the subjects that researchers have focused on in recent years, can help solve this problem. The ability of individuals to take, understand and apply instructions to make appropriate health decisions positively affects health outcomes (Buhr & Tannen, 2020; deWalt & Hink, 2009; Fry‐Bowers et al., 2014). For this reason, the integration of HL into the health system is emphasized in many countries (Sørensen et al., 2012; Vellar et al., 2016; Weishaar et al., 2019). HL is a condition that can be developed through education. Studies so far show that there is a relationship between HL and health outcomes (Dieng et al., 2020; Pawellek et al., 2022; Yoshii et al., 2020). However, there is only one study in the international literature examining the relationship between HL and fever management (Alqudah et al., 2019). This study provides a broad perspective on both fever management practices and HL levels of parents in this country.

## 2 BACKGROUND

Despite its prevalence and years of research, fever and its management in children cause anxiety in parents and are the leading cause of increased hospital admissions (Purssell & Collin, 2016). Fever occurs with the impaired balance in the thermoregulatory centre that regulates the body temperature due to reasons such as infection, fluid accumulation in the body, tissue damage and vaccination (Çaka et al., 2015; Yiğit & Sarıalioğlu, 2022). Fever, a symptom of many diseases in childhood, is a commonly encountered condition (Villarejo‐Rodríguez & Rodríguez‐Martín, 2019). Studies have reported that the rate of admission to primary care units and emergency departments in children with fever is between 24% and 30% (Piller & Herzog, 2019; Villarejo‐Rodríguez & Rodríguez‐Martín, 2019).

Fever is not a disease but rather a natural defence reaction of the body that facilitates recovery from diseases (Purssell & Collin, 2016; Villarejo‐Rodríguez & Rodríguez‐Martín, 2019). However, it is perceived as a disease by many families. Possible side effects that can be experienced due to fever (convulsions, brain damage, hearing loss and death) cause fear and anxiety in parents (Clericetti et al., 2019; Vicens‐Blanes et al., 2022; Wilson et al., 2019). Many parents perceive fever as harmful and believe that it should be treated (Vicens‐Blanes et al., 2022; Wilson et al., 2019). On the other hand, it is reported that unnecessary and wrong practices aimed at preventing fever are carried out due to fear, panic and anxiety (Komeagac & Bektas, 2018). Studies have reported that practices that may pose a danger to the child such as resorting to methods, including the use of water in the refrigerator, alcohol or vinegar to reduce fever, giving antibiotics and giving various and high doses of antipyretics, are carried out (Çaka et al., 2015; Gulcan & Sahiner, 2022; Thompson et al., 2020). The reason for this is that parents have insufficient basic knowledge about fever‐related decision‐making and caregiving (Gulcan & Sahiner, 2022; Thompson et al., 2020). It is possible to ensure that parents whose child has a fever manage this process in a healthy way by providing parents with interventional support addressing knowledge, practice and emotional areas (Kelly et al., 2016; Thompson et al., 2020). Another important issue that will positively affect fever management in cases where support cannot be provided or is insufficient is the high level of HL (Alqudah et al., 2019).

Nowadays, HL emerges as a key determinant for health promotion and development (Sarıyar & Kılıç, 2019; Sørensen et al., 2012). Hence, in many countries, the importance of education is emphasized by integrating HL into the healthcare system (Buhr & Tannen, 2020; Sørensen et al., 2012). This subject has attracted interest in many scientific fields and has become the focus of research (Firmino et al., 2018; Sørensen et al., 2012). HL is defined as ‘an individual's capacity to acquire, process, and understand basic health knowledge and services necessary to make appropriate health decisions’ (Fong et al., 2018). In other words, it contributes to acquiring a sufficient level of knowledge, awareness and ability to develop the right attitude and behaviour regarding health by individuals (Buhr & Tannen, 2020; deWalt & Hink, 2009; Fry‐Bowers et al., 2014). Considering the family dimension and especially the parent dimension, the importance of HL increases even more (Aral et al., 2021). Parents are responsible not only for their own life and health but also for the healthy life of their children (Buhr & Tannen, 2020). Parents use HL to meet their children's basic daily care and learn details about a health problem they experience (Pawellek et al., 2022). In the literature, it is known that a low level of HL leads to low self‐efficacy, inadequate health behaviour and negative health outcomes in parents (Dieng et al., 2020; Pawellek et al., 2022; Yoshii et al., 2020). Therefore, the development of parents' HL skills is important for the child's health.

Although HL has been a recently emphasized topic, to the best of our knowledge, there is limited evidence (Alqudah et al., 2019) that specifically considers the key aspects of parental HL and fever management. A study from Australia on this subject was found, but a study conducted in Turkish society was not encountered in the literature. A study was conducted from Australian parents with limited and functional HL. However, this study included a sample group representing each level of HL. Our sample of parents was larger than the sample used in the Australia study. We had the opportunity to compare and obtain more dimensional information with different HL scales. At the same time, our study also revealed differences in terms of cultural and regional fever management practices. We believe that these results will fill the gap in the literature.

## 3 METHODS

### 3.1 Aims

The primary aim of this study was to determine the relationship between the HL of Turkish parents and fever management of their children. Its secondary aim was to determine the practices of Turkish parents regarding the fever management of their children. Therefore, the study answers the following questions:

1. What are the practices of Turkish parents regarding the fever management of their children?
2. Is there a relationship between the HL of Turkish parents and fever management of their children?

### 3.2 Study design and sample

The present research is a cross‐sectional study. The present research was carried out in Sakarya province, located in the Marmara region of Turkey. It was conducted with 242 parents who applied to the paediatric emergency clinic of a hospital. The data were collected between September 2021 and September 2022. The Strengthening the Reporting of Observational Studies in Epidemiology (STROBE) statement was used in reporting the article.

The study population consisted of parents who applied to the paediatric emergency department between 08.00 and 17.00 for their child's fever complaint. To determine the sample size, a power analysis was performed using the G * Power (v3.1.7) program before starting the research. The minimum number of participants required to be statistically significant for correlation coefficient (*r* = .21) between the two scale scores was calculated as 230 mothers (*α* = .05, 1–*β* = .90). The sample comprised 242 parents who met the criteria for inclusion in the study, agreed to participate in the study voluntarily after being informed by the researchers, and filled out the forms related to the study completely. Only the mother or father of the child who was admitted with the complaint of fever was included in the study. Other inclusion criteria were as follows: (a) parents should be 19 years old and over, (b) they should speak and understand Turkish well, (c) they should volunteer to participate in the study, (d) the child with fever should be between 1 month and 5 years old, and (e) parents should not be healthcare professionals.

### 3.3 Data collection and ethical considerations

The aim of the study was explained to the parents who met the inclusion criteria. It was stated that the data would be protected safely. Verbal and written consents were obtained from the participants who volunteered to participate in the study. The interview with the parents who applied to the hospital for their child with fever took place after the children were given first aid. For the accuracy of the data, a moment when the children were calm and asleep was preferred. The data collection forms (Parent descriptive information form, Turkish Health Literacy Scale‐32 [THLS‐32] and Parents' Fever Management Scale [PFMS‐TR]) were given to the participant and requested to be filled out. The data collection forms were checked and received by the researchers. The data collection process took an average of 15–20 min. All stages of the study were carried out in accordance with the principles of the World Medical Association Declaration of Helsinki ‘Ethical Principles for Medical Research Involving Human Subjects’. The THLS‐32 was chosen because it was developed in line with the characteristics of Turkish culture.

Before starting the study, ethical approval was received from the Health Ethics Committee of Sakarya University, Sakarya, Turkey (Date: 30.06.2021 Number: 344). Written permission was obtained from the institutions. Verbal and written consents were acquired from the parents.

### 3.4 Measurement

In the study, data were collected using the Parent Descriptive Information Form, THLS‐32 and PFMS.

#### 3.4.1 Parent descriptive information form

The ‘Descriptive Information Form’ prepared by the researchers in line with the literature (Çaka et al., 2015; Thompson et al., 2020) to determine the descriptive characteristics of parents and children, consists of 28 questions. The descriptive characteristics of parents (age, education and employment status, economic status, family type, number of living children and chronic diseases) comprised 13 questions. There were also 15 questions that evaluated parents' approach to fever when the child had a fever. Additionally, this form contained a question. How worried are you about your child's fever? Parents were asked to answer this self‐reported question assessing their concerns as follows: 0 = not worried at all—10 = extremely worried.

#### 3.4.2 The Turkish health literacy scale

The scale was developed by Okyay et al. (2016). This scale was based on the conceptual framework developed by the European HL Survey Consortium. The scale which consists of 32 items in 5‐point Likert type, was developed to assess HL among literate people over the age of 15. THLS‐32 is structured as a 2 × 4 matrix, taking two basic dimensions (treatment and service, disease prevention and health promotion). The matrix consists of eight components, two dimensions and four processes (accessing health‐related information, understanding health‐related information, evaluating health‐related information, using/applying health‐related information). In the evaluation of the scale, the indices have been standardized on a scale of 0–50. The formula index = (mean‐1) × (50/3) was used for this. In this formula, the index is the index calculated unique to the individual and the mean refers to the mean of each item the individual responds to. Zero points received from the scale indicate the lowest HL, while 50 points indicate the highest HL. According to the scoring, HL is defined as follows; (0–25) points: insufficient HL, (26–33) points: problematic‐limited HL, (34–42) points: sufficient HL, and (43–50) points: excellent HL. In the validity findings of the scale, Kaiser–Meyer–Olkin (KMO) was found to be 0.908, and Bartlett's test was significant (χ^2^ = 5206.808 SD 496, *p* .001). Its variance is 54.754%. The construct validity of the scale was examined with the Extraction Method: Principal Components. It was observed that the factor loadings of the relevant items for the general scale and two dimensions were above 0.32 and were collected in a single factor. The overall internal consistency coefficient of the scale was found to be 0.927 (Okyay et al., 2016). Cronbach's alpha of the scale for the sample group was .94.

#### 3.4.3 Parents' fever management scale—Turkish version

It is used to assess parents' practices regarding childhood fever management. Altun et al. (2011) conducted the validity and reliability study of the original PFMS, developed by Walsh et al. (2008), in our country. The PFMS‐TR is a 5‐point Likert scale (1 = never, 2 = rarely, 3 = sometimes, 4 = mostly, 5 = always). It is one‐dimensional and consists of 8 items. The total score that can be obtained from the scale ranges from a minimum of 8 to a maximum of 40. Higher scores indicate higher fever management practices. Higher scores indicate more frequent or higher levels of these practices representing a higher parental burden when caring for a febrile child (Altun et al., 2011; Walsh et al., 2008). In the validity and reliability study, Cronbach's alpha of the scale was reported as .80 (Altun et al., 2011). Cronbach's alpha of the scale for the sample group was .73.

### 3.5 Data analysis

Statistical analyses were performed using the Statistical Package for the Social Sciences (SPSS) 22.0 package software. Number, percentage, mean, standard deviation were used for descriptive analyses. The Kolmogorov–Smirnov test was used to examine the normal distribution of the data. Pearson's correlation and multiple linear regression analyses were conducted to compare the total scale mean scores. There is no autocorrelation problem in the established model. The Durbin W value is between 1.5 and 2.5 (DW = 1.776) Cronbach's alpha value was used in the reliability analysis of the scale. The significance value was accepted as *p* < .05.

## 4 RESULTS

There were reached 297 parents within the scope of this study. Parents whose children had convulsions (*n* = 52) and who filled out the data collection tools incompletely (*n* = 5) were excluded from the study. The study was ended with 242 parents. Descriptive statistics of participants is given in 4.

### 4.1 Descriptive statistics

Of the parents participating in the study, 75.2% were mothers (*n* = 182), and 24.8% (*n* = 60) were fathers. The parents' mean age was 31.87 ± 6.59 (min 19–max 48). Concerning the distribution of parents according to their sociodemographic characteristics, it was found that 38.4% of the parents were high school graduates, 50.4% worked in a job, 55% lived in the city centre, 74.4% had an income equal to their expenses, and 84.3% had a nuclear family type. When the characteristics of the parents' children with fever were examined, it was determined that 51.2% were male, 8.7% had a previous history of convulsions, 85.5% had no chronic health problems, and 71.1% had been hospitalized for fever before.

### 4.2 Fever management practices of parents

Table 1 contains parents' practices regarding fever management (Table 1). Regarding the first practices, they did when their child had a fever, 61.2% of the parents stated that they took off their child's clothes. In this study, 21.9% of the parents defined fever as below 38°C and 18.5% as 39°C and above. In our study, the sources from which parents obtained information about fever were doctors (52.1%), nurses (30.2%), the internet (16.1%), neighbours–friends–relatives (7.4%), books–magazines–newspapers (6.2%) and radio‐television (1.2%), respectively.

**TABLE 1:** Distribution of parents' practices related to fever management (*N* = 242).

Parents' practices related to fever management	*n*	%
The first application when the child's fever rises^a^		
Take off the clothes of children	148	61.2
Take a warm shower	135	5.8
Give an antipyretic	125	51.7
Warm application/compress	63	26.0
Applying to a health institution	61	25.2
Cold application/compress	61	25.2
Make the child drink plenty of water	52	21.5
Cooling the room	27	11.2
Wipe with vinegar water	26	10.7
Wipe with alcoholic water	1	0.4
Wrapping child in blanket	1	0.4
Temperature measurement region		
Armpit	167	69.0
Forehead	59	24.4
Ear	12	5.0
Body	4	1.7
Frequency of measurement of temperature		
15 min apart	133	55.0
Half an hour apart	82	33.9
1 h apart	25	10.3
2 h apart	2	0.8
Fever limit specified by parents		
<38°C	53	21.9
Above 38°C	111	45.9
Above 38.5°C	33	13.6
Above 39°C	41	16.9
Above 39.5°C	2	0.8
Above 40°C	2	0.8
Training in fever management		
Yes	141	58.3
No	100	41.7
Source of information on the approach to the child with fever		
Doctor	126	52.1
Nurse	73	30.2
Written resources (Books, magazines, etc.)	15	6.2
Internet	39	16.1
TV/Radio	3	1.2
Neighbour, friend, close environment	18	7.4
Giving antipyretic		
According to doctor's prescription	134	55.4
According to the medicine given by the doctor when you have a fever before	84	34.7
According to the drug recommended by the pharmacist	3	1.2
What antipyretic is available at home?	21	8.7
Habit of reading the prospectus of drugs		
Yes	221	91.3
No	21	8.7
Frequency of admission to the emergency department due to fever		
First admission	33	13.6
Second admission	51	21.1
Third admission	52	21.5
Four or more admission	109	43.8

^a^ Participants marked more than one option.

### 4.3 Descriptive results and correlations between the health literacy and fever management

It was found that 10.7% of the parents participating in the study had an insufficient HL level, 40.9% had a problematic‐limited HL level, 35.1% had a sufficient HL level, and 13.2% had an excellent HL level. A significant difference was determined between HL levels and the PFMS‐TR (*F* = 8.281; *p* = .000) (Table 2). Table 2 presents the descriptive statistics of the scales and their correlations (Table 2).

**TABLE 2:** Descriptive results and correlations between the THLS‐32, PFMS‐TR and parent fever anxiety score (*N* = 242).

Scales		*n* (%)	PFMS‐TR	
THLS‐32	According to the rating health literacy levels		Descriptives	Test statistics
THLS‐32 score: 0–25 (1)	Insufficient health literacy level	26 (10.7)	34.61 ± 3.71	*F* = 8.281 *p* = .000 3 > 1^a^ 3 > 2^a^ 4 > 1^a^ 4 > 2^a^
THLS‐32 score: 26–33 (2)	Problematic‐limited health literacy level	99 (40.9)	35.42 ± 3.50	
THLS‐32 score: 34–42 (3)	Sufficient health literacy level	85 (35.1)	37.03 ± 3.20	
THLS‐32 score: 43–50 (4)	Excellent health literacy level	32 (13.2)	37.87 ± 2.62	

Scales	Mean ± SD	Min	Max	1	2	3
1. Parent fever anxiety score^b^	8.45 ± 2.05	1	10	1	*r* = −.004^c^ *p* = .955	*r* = .345^c^ *p* = .000**
2. THLS‐32	34.43 ± 7.79	11.6	50		1	*r* = .287^c^ *p* = .000**
3.PFMS‐TR	36.22 ± 3.46	8	40			1

*Note*: THLS‐32: The Turkish Health Literacy Scale, PFMS‐TR: Parents' Fever Management Scale—Turkish version. *F* = one‐way ANOVA.

^a^ Tukey ANOVA test.

^b^ That ise expresses concerns about their child's fever (0: Never worried −10: Extremely worried). Answers are based on parents' self‐report.

^c^ *r*: Pearson correlation coefficient.

** *p* < .05.

The total mean scores obtained by the parents from the scales were 34.43 ± 7.79 for the THLS‐32 and 36.22 ± 3.46 for the PFMS‐TR. The mean anxiety score of the parents when their child had a fever was found to be 8.45 ± 2.05. Considering the correlation analysis results, there was a statistically significant positive correlation between the PFMS‐TR and THLS‐32 (*r* = .287; *p* = .000). Furthermore, a statistically significant and positive correlation was detected between the parent fever anxiety score and PFMS‐TR (*r* = .345; *p* = .000). There was not a statistically significant correlation between the THLS‐32 and parent fever anxiety score (*r* = −.004; *p* = .955) (Table 2).

### 4.4 Power of the health literacy and the other variables to affect the fever management

According to the multiple linear regression analysis results, when the significance level corresponding to the *F* value is considered, it is observed that the established model is statistically significant (*F* = 21.487; *p* < .05). It is seen that 35.3% of the change in parents' fever management is explained by variables in Table 3 (Adjusted $R^{2}$=.353). Concerning the beta coefficient and *t*‐value of the independent variable and the significance level, the THLS‐32 has a statistically significant effect on the PFMS‐TR (*t* = 5.421, *p* < .05). A 1‐unit increase in the THLS‐32 variable causes an increase in .063 on the PFMS‐TR (*β* = .273). A 1‐unit increase in the parent fever anxiety score causes an increase in 0.493 on the PFMS‐TR (*β* = .292).

**TABLE 3:** Examination of factors affecting Parents' Fever Management Scale score (*N* = 242).

	Unstandardized coefficient		Standardized coefficient	*t*	*p*	95.0% CI	
	B	SE	Beta			Lower limit	Upper limit
(Constant)	63.763	12.189		5.231	.000	39.748	87.777
Parent fever anxiety score	0.493	0.091	.292	5.421	.000	0.314	0.672
THLS‐32	0.063	0.012	.273	5.159	.000	0.039	0.087
Living place	−1.426	0.380	−.205	−3.757	.000	−2.174	−0.678
Chronic health problems	−2.008	0.625	−.170	−3.214	.001	−3.239	−0.777
Training in fever management	1.196	0.375	.170	3.189	.002	0.457	1.934
Fever limit specified by parents	−0.843	0.313	−.144	−2.697	.008	−1.459	−0.227

*Note*: *F*: 21.348, *p* < .001, *R* ^2^: .353.

Abbreviations: B, unstandardized beta; CI, confidence interval; F, model statistic; *p*, significance level; PFMS‐TR, Parents' Fever Management Scale—Turkish version, *R* ^2^, correlation coefficient (Explained Variance Rate); SE, standard error; *t*, significance test of regression line; THLS‐32, The Turkish Health Literacy Scale; *β*, standardized beta *β*.

## 5 DISCUSSION

In this section, it was aimed to discuss parents' practices regarding the fever management of their children and the findings showing the relationship between their HL and fever management of their children in line with the literature. The concept of our study is new to Turkish society.

Considering the first practices done when the child's fever rises, it was found that the parents took off their child's clothes in the first place. It was determined that they gave antipyretics, did warm application/compress and preferred to apply to a health institution, respectively. Likewise, previous studies found that the most preferred method was to take off the clothes of children (Akbayram, 2021;Göbekli & Güney, 2022; Gulcan & Sahiner, 2022). These practices are the practices specified in the evidence‐based guidelines (Chiappini et al., 2017; Paul et al., 2022). Giving antipyretics was reported as the first choice in this studies (Hew et al., 2019). Furthermore, it was revealed that a small group of parents used non‐medical practices to reduce fever, such as wiping with water containing alcohol (0.4%) and wiping with water containing vinegar (10.7%). In recent studies, the rate of wiping with water containing vinegar to reduce fever was reported as 11.9% by Gulcan and Sahiner (2022), 12.9% by Göbekli and Güney (2022) and 4.8% by Yiğit and Sarıalioğlu (2022). These rates are close to each other. Worryingly, alcohol was used to reduce fever, although the rate was low. It is known that alcohol is a method that cannot be recommended for reducing fever and even causes alcohol poisoning due to inhalation by the child (Chiappini et al., 2017). The fact that these methods are still used despite the recommendations of fever management guidelines (Chiappini et al., 2017; Paul et al., 2022) indicates a knowledge gap between what is taught and what parents practice.

In this study, 69% of the parents indicated the armpit as the temperature measurement body part. It is stated in the literature that it is the most frequently used body part and its usage rate varies between 21.1 and 76.9 (Göbekli & Güney, 2022; Gulcan & Sahiner, 2022; Morris et al., 2021; Vicens‐Blanes et al., 2022; Yiğit & Sarıalioğlu, 2022). These rates are consistent with the study findings. In the studies by Salih et al. (2022) and Yiğit and Sarıalioğlu (2022), temperature measurement from the forehead was in the first place (Salih et al., 2022; Yiğit & Sarıalioğlu, 2022). Although it was included in the questionnaire, it was found that parents never used the rectal and oral body part for temperature measurement. In other studies conducted in our country, the rate of using these regions by parents is low (Göbekli & Güney, 2022; Gulcan & Sahiner, 2022). The reason why the armpit area is preferred may be the ease of measurement from the area and safer measurement.

In studies defining fever, temperatures between 36.0 and 37.9°C were considered normal temperature, temperatures above 38.0°C were considered fever, and temperatures above 40°C were considered high fever (Salih et al., 2022; Walsh et al., 2008). According to this information, the study determined that 21.9% of the parents defined the temperature, which was not defined as fever, as fever. Likewise, 18.5% reported that they defined a temperature of 39°C and above as fever. These findings were remarkable. The proportion of parents who misidentified fever is similar to the findings of other studies (Gulcan & Sahiner, 2022; Hew et al., 2019; Zyoud et al., 2013). This inaccurate information may result in unnecessary practices or medication intervention by parents or, on the contrary, delays in determining the severity of the illness in a febrile state.

Four out of five parents reported that they received information from the health professional about the approach to the child with fever. This result is pleasing. In other studies, similar to our study results, it was stated that parents received information about fever management from health professional, primarily doctors and then nurses, at the highest rate (Göbekli & Güney, 2022; Kelly et al., 2017; Thompson et al., 2020). Although the order differs from the study, it is followed by receiving information from the internet, television, neighbours, and friends (Yiğit & Sarıalioğlu, 2022). In our study, the rate of receiving information from neighbours, friends and close circles was 7.4%, and the rate of receiving information from the internet was 16.1%. This rate is not low. Often the information acquired from the close circles is not evidence‐based information. There may be a risk of harm to the child (Monsma et al., 2015). Therefore, it should be ensured that accurate information is obtained from the right source and is accessible to parents.

Although there are racial/ethnic differences in beliefs and practices related to childhood fever, the feelings experienced are the same (Yiğit & Sarıalioğlu, 2022). The study found that the fever anxiety score of the parents was quite high (8.45). Studies in different cultures determined that parents experienced feelings of anxiety, fear and panic intensely when their children had a fever (Göbekli & Güney, 2022; Kerdar et al., 2021). It is stressed that parents' anxiety leads to wrong or inappropriate practices (Zyoud et al., 2013). It is important for parents to have low anxiety about fever so that they can manage fever correctly (Yiğit & Sarıalioğlu, 2022). The anxiety of parents may be related to the side effects of fever, parenting role and loss of control.

The use of antipyretics is associated with higher levels of anxiety (Kerdar et al., 2021). In our study, 55.4% of the parents gave medication according to the doctor's prescription. However, the other half reported that they gave medication without consulting a doctor. In another study carried out in our country, the rate of giving medication according to a doctor's prescription was 19.4% (Yiğit & Sarıalioğlu, 2022). In the study by Sakr et al. (2022) more than 75% gave their children an antipyretic, often without consulting a doctor (Sakr et al., 2022). This indicates that over‐the‐counter medication use is widespread. These data clearly demonstrate the need to educate parents on appropriate fever management strategies and preventing wrong medication and overdose use.

In the study, the mean PMFS‐TR score of the parents was 36.22. In studies from Turkey, the mean PMFS‐TR score was reported as 33.71 (Göbekli & Güney, 2022), 33.39 (Gulcan & Sahiner, 2022), 33.54 (Cinar et al., 2014) and 33.44 (Yiğit & Sarıalioğlu, 2022). In studies conducted in Australia, the total score obtained by parents from the scale was 17.2 out of 35 (Walsh et al., 2008), and it was reported to increase to 24.8 over the years (Wilson et al., 2019). The results of this study and studies in the literature are similar, indicating high fever management practices.

HL, or the ability to read and understand health information, is crucial to children's health (Alqudah et al., 2019; Pawellek et al., 2022). In the present study, the parents' mean THLS‐32 total score was 34.43. It was found that 10.7% of the parents had an insufficient HL level, 40.9% had a problematic‐limited HL level, 35.1% had a sufficient HL level, and 13.2% had an excellent HL level. In many studies, to facilitate evaluation and comparison, scores below 33 were evaluated as insufficient, and scores of 33 and above as sufficient. In this regard, our study determined that about half of the parents had sufficient HL and half had insufficient HL. In the study conducted in Turkey in 2014 within the scope of the European HL Project, the HL index was found to be 30.4 (Tanrıöver et al., 2014). In previous studies, the HL levels of participants were insufficient (Brandstetter et al., 2020; Özdemir et al., 2020; Phommachanh et al., 2021; Sørensen et al., 2012).

HL is required to understand and evaluate fever, read medication labels, calculate the dose, measure medications and understand information about fever management (Monsma et al., 2015). Our study determined that with an increase in the HL level of parents, fever management of their children increased significantly. There was only one study that evaluated the relationship between these two subjects. The study by Alqudah et al. (2019) on children and parents who applied to the emergency department found no difference in terms of low HL and high literacy and parents' fever knowledge or fever management. The knowledge and skills related to fever management were stated to be low in both groups. However, although there was no significant difference, the researchers underlined that the lack of knowledge should be eliminated and the level of HL should be increased (Alqudah et al., 2019). Likewise, this information confirms the results of a recent systematic review (Batool et al., 2024). Another systematic review reported that low HL was associated with poorer health knowledge and disease management, increased chronic diseases, inadequate use of preventive health services and increased hospitalizations (Zaidman et al., 2023). It is thought that a better understanding of health‐related information plays an important role in controlling fever management.

Parents' HL is a factor that affects health behaviours towards their children (Pawellek et al., 2022). In our findings, we revealed that 8.2% of the change in parents' fever management was explained by HL. It is known that the correct knowledge, perception, attitude and skills are effective in acquiring healthy behaviours. This result indicated the improvement of parents' HL levels in fever management. It also clearly reveals that parents should be educated immediately, considering the importance of parents' HL and its effect on a child's health.

### 5.1 Study limitations

The study has some limitations. The findings are limited to the participants' self‐reports, and the data were obtained from a single centre and cannot be generalized. The measured situations are limited to the scale items. The THLS‐32 measures the general HL level of parents. Therefore, the HL measurement of parents regarding fever and fever management could not be evaluated. This is the last limitation of the study. To the best of our knowledge, the present study is the first to demonstrate the effect of HL on childhood fever management in our society. Another strength is that both mothers and fathers formed the sample of the study to understand the fever management practices in the family.

## 6 CONCLUSION

The parents' mean PMFS‐TR score was 36.22, and their mean THLS‐32 total score was 34.43. The results acquired from the study (i) showed that half of the parents had a problematic or insufficient HL level and (ii) indicated that parents had high fever management practice and care burden when their children had a fever. Results shows a lack of knowledge and wrong practices among parents in terms of the practices for children with fever in Turkish society. The study revealed that with an increase in the HL level of parents, fever management for their children increased significantly. It was observed that 8.2% of the change in parents' fever management was explained by HL. In this context, increasing the HL level of parents can be a long‐term positive acquisition in both fever management and other child health issues. There is a need for sustainable educational interventions in the societal context to eliminate misconceptions about childhood fever and its management. health professionals should expand the framework of the training they offer to parents and increase the degree of impact. It is recommended to develop standard approaches and guidelines for correct and safe fever management. Future studies should investigate the feasibility of an intervention to help parents effectively manage their child's fever, considering their HL level. Moreover, it is recommended to develop measurement tools that evaluate the HL levels of parents regarding childhood fever and fever management. It should be a basic nursing skill that to provide HL support to parents.

## AUTHOR CONTRIBUTIONS

**Dilek Menekşe:** Conceptualization; methodology; investigation; data curation; formal analysing; writing—original draft preparation; writing—reviewing and editing. **Öznur Tiryaki:** Methodology; data curation; formal analysing; writing—reviewing and editing. **Nursan Çınar:** Methodology; writing—reviewing and editing.

## FUNDING INFORMATION

This research received no specific grant from any funding agency in the public, commercial or not‐for‐profit sectors.

## CONFLICT OF INTEREST STATEMENT

No conflict of interest has been declared by the authors.

### PEER REVIEW

The peer review history for this article is available at https://www.webofscience.com/api/gateway/wos/peer‐review/10.1111/jan.16275.

## ETHICS STATEMENT

Ethical approval was received from the Health Ethics Committee of Sakarya University, Sakarya, Türkiye (Date: 30.06.2021 Number: 344).

## CONSENT TO PARTICIPATE

Informed consent was obtained from all individual participants included in the study.

## CONSENT FOR PUBLICATION

Not applicable/no identifying information was used for any individual participant.

## Supporting information


Data S1:

## Data Availability

The data that support the findings of this study are available on request from the corresponding author. The data are not publicly available due to privacy or ethical restrictions.
